# Supplementary material for: A register-based cohort study on the effectiveness and Safety of anti-PCSK9 treatment in persons with hyperlipidemia
Source: Commun Med (Lond). 2024 Oct 7;4:193. doi: 10.1038/s43856-024-00611-x (PMC11458803; doi:10.1038/s43856-024-00611-x)
Supplement: Supplementary file 2 — Supplementary Information [file 43856_2024_611_MOESM2_ESM.pdf]

SUPPLEMENTARY INFORMATION

A register-based cohort study on the effectiveness and Safety of anti-PCSK9 treatment in persons with hyperlipidemia

Authors: Michael Asger Andersen1, Anne Helms Andreasen2, Lia Evi Bang3, Espen Jimenez Solem1, and Tonny Studsgaard Petersen1

1 Department of Clinical Pharmacology, Copenhagen University Hospital - Bispebjerg and Frederiksberg Hospital, Copenhagen, Denmark

2 Center for Clinical Research and Prevention, Copenhagen University Hospital - Bispebjerg and Frederiksberg, Copenhagen, Denmark

3 Department of Cardiology, Copenhagen University Hospital - Rigshospitalet, Copenhagen, Denmark

Content

Supplementary Methods 1

Supplementary Methods

WebI

SAP Web Intelligence (WebI) is a tool that is integrated into the EPIC electronic medical record system. It allows users to create custom reports and analyses of data stored in the EPIC Clarity database. With WebI, users can build queries using a wide range of predefined variables and filters. WebI and can generate reports in a variety of formats including tables, graphs, and maps.

Mgcv

In the present study, we used generalized additive models (GAMs) as implemented in the mgcv package in R to analyze the data. GAMs are a flexible class of regression models that allow for the incorporation of non-linear relationships between the response variable and predictor variables. They are particularly useful for modeling complex, non-linear trends in data and can be seen as an extension of linear regression models. The mgcv package is a popular and widely used tool for fitting GAMs, and it provides a range of options for specifying and estimating model parameters. Overall, the use of GAMs and the mgcv package allowed us to accurately model and analyze the data, leading to insights and conclusions that would not have been possible with traditional linear regression models. To check the model fit of a GAM fitted with the mgcv package, the gam.check() function can be used. This function produces a plot that displays the residuals of the model, as well as a number of diagnostic plots that can be used to assess the model fit. The gam.check() function produces a plot that displays the residuals of the model on the left hand side, and a number of diagnostic plots on the right hand side. The residuals plot shows the residuals of the model as a function of the fitted values. Ideally, the residuals should be randomly distributed around zero, with no discernible pattern. If there is a pattern present in the residuals plot, it may indicate that the model is not adequately fitting the data. In mgcv, most smooths are required to satisfy a sum-to-zero identifiability constraint, which allows for the inclusion of an intercept in the model, especially when the model includes factor parametric terms. This constraint centers the smooth around 0 on the axis. The 0 line represents the average value of the response (or the reference levels for factor parametric terms) on the link scale.

Negative values on the axis indicate that the effect of the covariate causes the response to be below average, while positive values indicate that the response is above average, all while taking into account the other estimated model terms.

Definitions

In this study, we used the International Classification of Diseases (ICD) Tenth Revision (ICD-10) codes, the Anatomical Therapeutic Chemical (ATC) codes, and the Nomenclature for Properties and Units (NPU) codes to categorize diagnoses, conditions, medications, and laboratory measurements, respectively. ICD-10 codes are standardized codes used for medical billing and record-keeping purposes. They are used to assign unique codes to every diagnosis, symptom, and procedure recorded in healthcare settings. ATC codes, on the other hand, are a classification system for medications, developed by the World Health Organization (WHO). They are used to classify drugs according to their therapeutic, pharmacological, and chemical characteristics. NPU codes, meanwhile, are used for assigning laboratory measurements. In this study, we used these codes to classify diagnoses and conditions, medications. The use of ICD-10, ATC, and NPU codes allowed us to accurately and consistently record and analyze the data from the laboratory, ensuring the reliability and validity of the results.

Atherosclerotic cardiovascular disease (ASCVD):

Disease	Diagnostic code:	Operation code:
Coronary heart disease (CHD)	I20, I21, I23, I24 and I25	KFNA, KFNB, KFNC, KFND, KFNE, KFNH20, KFNF, KFNG00, KFNG02, KFNG05 and KFNG30
Atherosclerotic cerebrovascular disease	I630, I631, I632, I633, I634, I635, I638, I639, I66, I672, G458 and G459	KAAL10 and KAAL11
Peripheral artery disease (PAD)	I65, I70, I713, I714, I715, I716, I739A, I739C, I741B, I742B, I743B, I744B, I745B, K550H and K551A	KPAF21, KPAK21, KPAF20, KPCE, KPCF, KPCH, KPCN, KPCP, KPDE, KPDF, KPDH, KPDN, KPDP, KPEE, KPEF, KPEH, KPEN, KPEP, KPFE, KPFF, KPFH and KPFN

Hypercholesterolemia criteria:

Disease:	Diagnostic code:
Hypercholesterolemia	E780A, E780C, E780D, E780E, E781, E782, E783, E784 and E785

Familial hypercholesterolemia (FH):

Disease	Diagnostic code:
FH (non-specific)	E780B
Homozygote FH	E780B2
Heterozygote FH	E780B1

Procedures prior to inclusion:

Procedure:	Procedure code:	Operation code:

Percutaneous coronary intervention (PCI)	None	KFNG and KZFFX01
Coronary artery bypass grafting (CABG)	None	KFNA, KFNB, KFNC, KFND, KFNE, KFNH20 and KFNF
Angioplasty and stent placement	None	KPAN, KPAP, KPBN, KPBP, KPCN, KPCP, KPDN, KPDP, KPEN, KPEP, KPFN and KPFP
Endarterectomy	None	KPEF, KPAF, KPBF, KPCF and KPDF
Thrombectomy	None	KAAL11, KFAB10, KPAE10, KPAE25, KPAE30, KPAE99, KPAU74, KPBE10, KPBE20, KPBE30, KPBE99, KPCE30, KPCE40, KPCE99, KPDE10, KPDE30, KPDU74, KPEE10, KPEE11, KPEE12, KPFE10, KPFE30, KPHE22, KPHE23, KPHE25, KPHE30, KPHE31 and KPHE99

Comorbidities prior to inclusion:

Cardiovascular comorbidities:

Disease:	Diagnostic code:	Operation code:	ATC-code:
Diabetes (DM)	E10, E11 and N083	None	A10
Diabetes target organ damage	H360, E103, E113, G590, E104, E114, N083, E102 and E112	None	None
Chronic kidney disease (CKD)	E102, E112, I120, N03, N04, N05, N06, N07, N08, N11, N12, N162, N163, N164, N182, N183, N184, N185, N26 and Q61	None	None
Chronic obstructive pulmonary disease	J43 and J44	None	None
Hypertension	I10, I11, I12, I13 and I15	None	C07, C08, C09, C03A, C03B, C03C, C03EA, C03EB, C02L, C10BX, C03DA, C03DB, C03E, C02DB, C02DC, C02DD, C02DG, C02A, C02B and

			C02C (excluding C02AC02)
Heart failure	I099A, I110, I130, I132, I420 and I50	None	None
Cancer, ex. non-melanoma skin cancer	C00, C01, C02, C03, C04, C05, C06, C07, C08, C09, C10, C11, C12, C13, C14, C15, C16, C17, C18, C20, C21, C22, C23, C24, C25, C26, C30, C31, C32, C33, C34, C37, C38, C39, C40, C41, C43, C45, C46, C47, C48, C49, C50, C51, C52, C53, C54, C55, C56, C57, C58, C60, C61, C62, C63, C64, C65, C66, C67, C68, C69, C70, C71, C72, C73, C74, C75, C76, C77, C78, C79, C80, C81, C82, C83, C84, C85, C86, C88, C90, C91, C92, C93, C94, C95 and C96	None	None
Liver disease	K70, K71, K72, K73, K74, K75, K76, K77, B15, B16, B17, B18, B19 and B581	None	None
Rheumatoid arthritis	M05, M060, M069 and M080	None	None

Laboratory values:

Biomarker:	Laboratory code:
Low-density lipoprotein cholesterol (LDL-C)	NPU01568, NPU10171, AAB00101, AAB00102 and DNK35308
High-density lipoprotein cholesterol (HDL-C)	NPU01567 and NPU10157
Total cholesterol (TC)	NPU18412 and NPU01566
Triglycerides (TGs)	NPU04094 and NPU03620
Lipoprotein a (Lp[a])	NPU21687, NPU19840 and NPU58475
Apolipoprotein B (Apo-B)	NPU22299 and NPU19697

Cholesterol-lowering drug group classification:

Contains the ATC-codes used to define the cholesterol-lowering drug groups used in multiple endpoints.

Drug group:	ATC-code:
Statin	C10AA, C10BA01, C10BA02, C10BA03, C10BA04, C10BA05, C10BA06, C10BA07, C10BA08, C10BA09 and C10BX
PCSK9i	C10AX13 and C10AX14
Ezetimibe	C10AX09, C10BA02, C10BA05, C10BA06 and C10BA10
Bile acid sequestrants	C10AC01, C10AC02, C10AC03 and C10AC04
Fibrates	C10AB, C10BA03, C10BA04 and C10BA09
